# A meta-analysis of the effect of binge drinking on the oral microbiome and its relation to Alzheimer’s disease

**DOI:** 10.1038/s41598-020-76784-x

**Published:** 2020-11-16

**Authors:** Ayuni Yussof, Paul Yoon, Cayley Krkljes, Sarah Schweinberg, Jessica Cottrell, Tinchun Chu, Sulie L. Chang

**Affiliations:** 1 Department of Biological Science, Seton Hall University, 400 S Orange Ave, South Orange, NJ 07079 USA; 2 The Institute of NeuroImmune Pharmacology (I-NIP), Seton Hall University, 400 S Orange Ave, South Orange, NJ 07079 USA

**Keywords:** Neuroscience, Microbiology

## Abstract

The diversity of bacterial species in the oral cavity makes it a key site for research. The close proximity of the oral cavity to the brain and the blood brain barrier enhances the interest to study this site. Changes in the oral microbiome are linked to multiple systemic diseases. Alcohol is shown to cause a shift in the microbiome composition. This change, particularly in the oral cavity, may lead to neurological diseases. Alzheimer’s disease (AD) is a common neurodegenerative disorder that may cause irreversible memory loss. This study uses the meta-analysis method to establish the link between binge drinking, the oral microbiome and AD. The QIAGEN Ingenuity Pathway Analysis (IPA) shows that high levels of ethanol in binge drinkers cause a shift in the microbiome that leads to the development of AD through the activation of eIF2, regulation of eIF4 and p70S6K signaling, and mTOR signaling pathways. The pathways associated with both binge drinkers and AD are also analyzed. This study provides a foundation that shows how binge drinking and the oral microbiome dysbiosis lead to permeability changes in the blood brain barrier (BBB), which may eventually result in the pathogenesis of AD.

## Introduction

The Human Microbiome Project, launched in 2007, is a research initiative by the National Institute of Health (NIH) with the goal of identifying and characterizing the human microbiome (HM). HM is defined as all the bacteria, viruses, and fungi that reside on and in the human body^1,2^. These microflora in the body play an important role in helping the body perform tasks like controlling the immune system, metabolic function, protecting against harmful bacteria, digesting fiber and neurological function^3,4^. The microbiome is a key part of biological function that challenges the concept of organism individuality as it has been discovered that the microbial community is at least as abundant as our somatic cells, but the diversity in genes is far greater than the human genome^5^. Current research shows only 10% of our genes are truly derived from human hosts while the rest comes from the microbiota that commensally resides on and in us^6^. Advancements in technology and cost reduction of sequencing have led to an increase in profiling of the human microbiome through 16S rRNA gene taxonomic, metagenome and meta-transcriptome analysis^7,8^. The increase in microbiome profiling has led to a shift in how the microbiome is studied. Instead of just profiling the microbiome, there is now an emphasis on looking into a more comprehensive study of understanding how the bacteria community affects the host’s health.

The Human Oral Microbiome Database (HOMD) has been created with the goal of providing the scientific community with compiled and collaborative information on the species of bacteria that are present in the mouth^9^. Based on HOMD, approximately 700 prokaryotes taxa reside in the human oral cavity, but most are still uncharacterized due to the inability to culture the species^10^. This diversity in species also depends on the microenvironment within the oral cavity which includes the teeth, tongue, hard and soft palates, tonsils, and gingival sulcus^10^. The diversity in bacteria is seen in every aspect of microenvironment, such that one milliliter of saliva contains an average of 1.4 × 10^4^ cells that contain seven major phyla, which are *Fusobacteria, Firmicutes, Bacteroides, Actinobacteria, Proteobacteria, Spirochaetes* and *Saccharibacteria* (formerly known as TM7)^6^.

A key interest in microbiome study is to establish the connection between the microbiome and different diseases. The oral microbiome has specifically been shown to be involved in the pathogenesis of dental caries, cancer, gastrointestinal disorder, nervous system disorder, endocrine disorder, cardiovascular disorder, and immune system disorder^11–13^. The crucial link between the microbiome and diseases occurs both in normal and disease flora, making it important to establish this link between diseases and the microbiome. The link between the gut microbiome and the central nervous system (CNS), for example, is one of the most widely-studied connections^14–16^. A greater focus should be placed on establishing the correlation between the oral microbiome and CNS diseases as current research shows a possible link between the oral microbiome and Alzheimer’s Disease (AD), but the exact influence is still being studied^17,18^.

Despite the fact that alcohol metabolism takes place mainly in the liver, studies have found that the level of a byproduct of alcohol metabolism, acetaldehyde, is higher in saliva than in the blood immediately after alcohol consumption while the concentration of alcohol in the saliva is equivalent to the blood alcohol within thirty minutes of consumption^19^. This leads to the accumulation of toxic molecules like acetaldehyde that damage the mucosal and glandular tissues^20^. The damaged mucosa causes an impairment of the immune function that may lead to microbial infection and changes to the oral microbiome^20^.

According to the National Institute on Alcohol Abuse and Alcoholism (NIAAA), the average alcohol content of beer is about 4.5% while wine contains 12.9% alcohol and spirits contain 41.1% alcohol^21^. Based on the percentage of alcohol, one standard drink is about 0.5 fl oz of alcohol, which is equivalent to 12 fl oz of regular beer, 5 fl oz of wine, and 1.5 fl oz of 80-proof distilled spirits^21^. One standard drink will raise blood alcohol concentration (BAC) by 0.02%^22^. Binge drinking is defined as consuming four or more drinks for women and five or more drinks for men on one occasion, or a BAC of 0.08% (NIAAA). People between 18–20 and 21–25 years of age have the highest rates of binge drinking activities^23^.

AD is a neurodegenerative disorder and the leading cause of dementia in the elderly^24^. The amyloid cascade hypothesis suggests that AD is a result of the body’s generation of amyloid beta (AB) being greater than its clearance. The accumulation of AB is thought to eventually cause neurodegeneration^25^. Generally, the brain is impermeable due to the tight junctions formed within the blood–brain barrier (BBB). However, the circumventricular system is a portion within the brain that allows substances to pass through that otherwise would not^26^. A weakened BBB may allow bacteria to enter the brain and influence the pathogenesis of AD^27^.

This meta-study aims to look at the combination of effects of binge drinking, the oral microbiome and their effects on the pathogenesis of AD. This study establishes the link between how the effects of binge drinking and dysbiosis of the oral microbiome cause changes in the membrane permeability of the BBB, which eventually leads to the development of AD. Finding the links between the oral microbiome, alcohol and AD can help us further understand the development of AD and ways that it can be treated.

## Result

### Oral microbiome of binge drinkers

**Figure 1:**
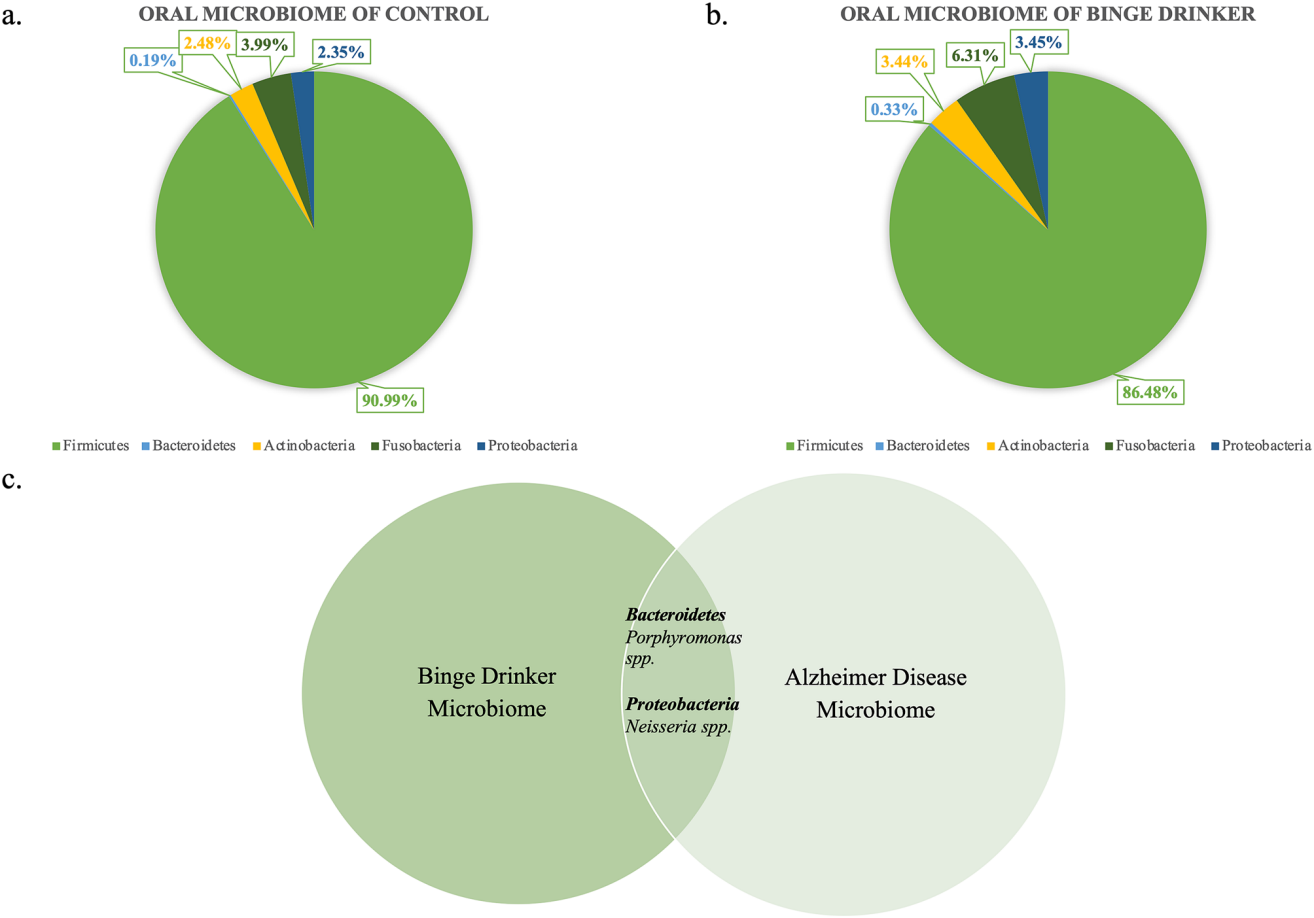
Microbiome comparison of different conditions. (**a**) The composition of the oral microbiome of controlled individuals. (**b**) The composition of the oral microbiome of binge drinkers. (**c**) A Venn Diagram comparing the overlapping species between binge drinker’s oral microbiome and AD oral microbiome.

The oral microbiome is not as well-characterized as the widely-studied gut microbiome. This is based on the HMP data, which indicates 229 datasets are available for gastrointestinal tract versus 26 datasets for the oral cavity. The data in Fig. 1 has been obtained from large studies of American adults^28^ and the information obtained from Disbiome Database^29^. The microbiome profiles of controlled non-drinkers (n = 270) and binge drinkers (n = 160) have been characterized and summarized in the pie charts in Fig. 1a,b. The consumption of alcohol shows a shift in the oral microbiome of binge drinkers compared to the control. Binge drinking leads to the inhibition of the *Firmicutes* phyla, which is reduced from 90.99 to 86.48%. The most significant proliferation in the presence of alcohol has been observed in the *Fusobacteria* phyla, which has increased from 3.99 to 6.31%. The other three phyla, *Bacteroides, Actinobacteria* and *Proteobacteria*, have also seen an increase in proliferation in binge drinkers in comparison with the control. Figure 1c shows the overlapping key genus between the binge drinkers’ oral microbiome and the AD microbiome. There are 23 key genera and species that have been linked to AD. Based on the *Firmicutes* phyla*,* the key genera are *Dialister, Clostridium, Turicibacter, Blautia, Phascolarctobacterium* and *Gemella*. There are 5 genera that have been noted as significant for *Bacteroidetes* phyla*: Eubacterium, Bacteroides, Alistipes, Porphyromonas* and *Eubacterium*. As for *Actinobacter* phyla, there are 4 key genera: *Bifidobacterium, Corynebacteriaceae, Propionibacteriaceae* and *Adlercreutizia*. As for *Proteobacteria* phyla, there are seven key genera that have been linked to AD*: Shigella, Salmonella, Klebsiella, Neisseria, Escherichia* and *Bacteroides.* Finally, for *Fusobacteria* phyla*,* only *Fusobacterium* has been noted to be significant to AD. In terms of the overlapping species, the two key species that are found in both binge drinkers and AD are *Porphyromonas* spp. from *Bacteroidetes* phyla and *Neisseria* spp. from *Proteobacteria* phyla*.*

### Canonical pathway of binge drinker

**Figure 2:**
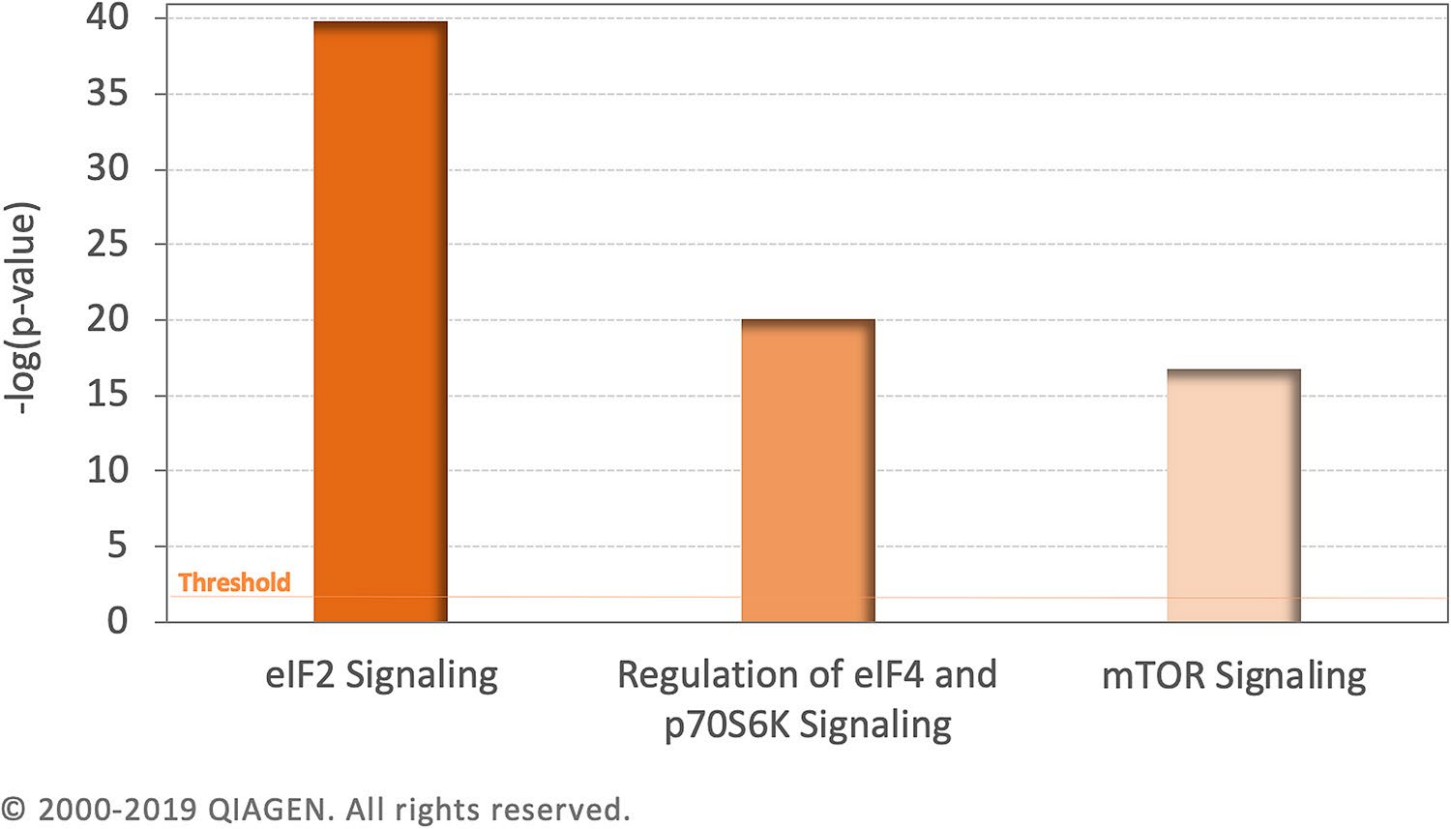
Top 3 pathways affected in binge drinkers. The top 3 canonical pathways affected in the sample are eIF2 Signaling, Regulation of eIF4 and p70S6K Signaling and mTOR signaling. The orange line represents the threshold with a cutoff *p* value of 0.05.

Figure 2 shows the top 3 canonical pathways of binge drinkers that have been analyzed via Core Analysis in IPA. For − log (*p* value) greater than 16, the analysis has shown 85 pathways are activated in the dataset. The top pathway is Eukaryotic Initiation Factor 2 (eIF2) with 59 affected, Z-score of 5.477, − log(*p* value) of 39.747 and *p* value of 1.79E−40. The second pathway is Regulation of eIF4 and p70S6K Signaling with 34 molecules affected, Z-score of 0.816, − log(*p* value) of 20.027 and *p *value of 9.40E−21. The third most significant pathway is the mammalian target of rapamycin (mTOR) pathway with 35 molecules affected, Z-score of 1.897, − log(*p* value) of 16.775 and *p* value of 1.68E−17. The data has also predicted the activation of AD to be *p* value of 1.14E−11 and Z-score of 1.89. The orange line shows the cut for significant value at *p* value of 0.05.

### eIF2 pathway in binge drinker

**Figure 3:**
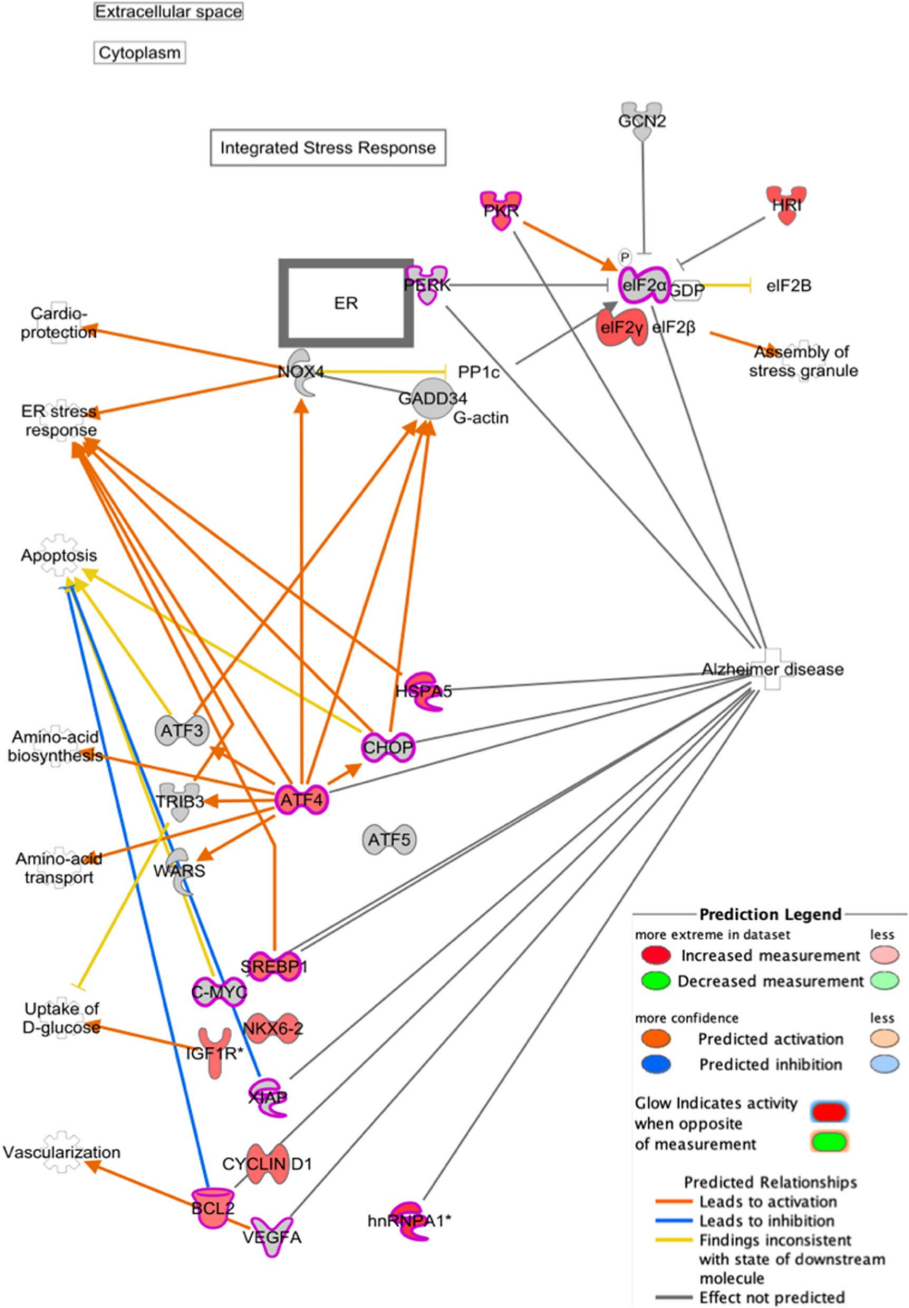
EIF2 pathway overlay with the experimental data, AD detection and molecules activation prediction (MAP). The red-filled molecule indicates that it is expressed in the dataset, grey-filled molecule is not detected but involved in eIF2 pathway and pink outline indicates association with AD in IPA knowledge base. The orange line indicates activation; the blue line indicates inhibition; and the yellow line indicates inconsistent findings while the grey line indicates no prediction.

Figure 3 shows the IPA curated information of AD through the eIF2 pathway. Twelve molecules, outlined in pink, are noted to be linked to AD while 11 molecules, which are colored in red, are detected in the dataset. The grey molecules are involved in the pathway but not detected in the dataset. One of the main components of eIF2 involved in the pathogenesis of AD is eukaryotic initiation factor 2 alpha (eIF2α). In the cell cytoplasm, the double stranded RNA-activated protein kinase or eukaryotic translation initiation factor 2 alpha kinase 2 (EIF2AK2 or PKR) increases inhibition of phosphorylated eIF2α^30^.

In the pathway, upstream kinase phosphorylates eIF2α, which prevents the recycling and inhibition of translation initiation from normal capped mRNA^30^. The phosphorylation of eIF2α subunits by various kinase such as eukaryotic translation initiation factor 2 alpha kinase 3 (EIF2AK3 or PERK) and EIF2AK2 (PKR). PERK is activated via the accumulation of misfolded proteins, or endoplasmic reticulum (ER) stress. When PERK phosphorylates eIF2α, it reduces the ER stress by preventing translation initiation and allows the ER to refold the misfolded proteins or degrade them^30^. The data has shown an overexpression of PKR, which leads to the phosphorylation of eIF2α. The accumulation of eIF2α-p inhibits eIF2β, thus blocking the exchange of guanosine diphosphate (GDP) to guanosine triphosphate (GTP).

Overall, the most significant association has been found through the phosphorylation of eIF2α via PKR and ATF4. Phosphorylated eIF2α is significantly increased in the brains of AD patients. The accumulation of misfolded proteins such as Amyloid-β can activate eIF2α phosphorylation. When phosphorylated, eIF2 becomes a competitive inhibitor of eIF2β by blocking the exchange of GDP to GTP. Although eIF2 phosphorylation inhibits global protein synthesis, it also translates a subset of mRNAs that contains upstream open reading frames^30^. mRNAs that can undergo translation are β-secretase enzyme (BACE1) and the CREB repressor, activating transcription factor 4 (ATF4). Both have been associated with the AD pathology. Increased levels of BACE1, although not shown in the figure, has been associated with β-amyloidgenesis. BACE1 cleaves amyloid precursor protein and releases the membrane-bound C terminal fragment. Further cleavage through γ-secretase results in Aβ proteins, a hallmark of AD. ATF4 mRNA translation is also selectively induced when eIF2α is phosphorylated. ATF4 overexpression has been shown to facilitate oxidative stress-induced cell death^31^. Overexpression of ATF4 has also been observed within the brains of AD patients^32^.

### Relationship of AD, BBB and LPS

**Figure 4:**
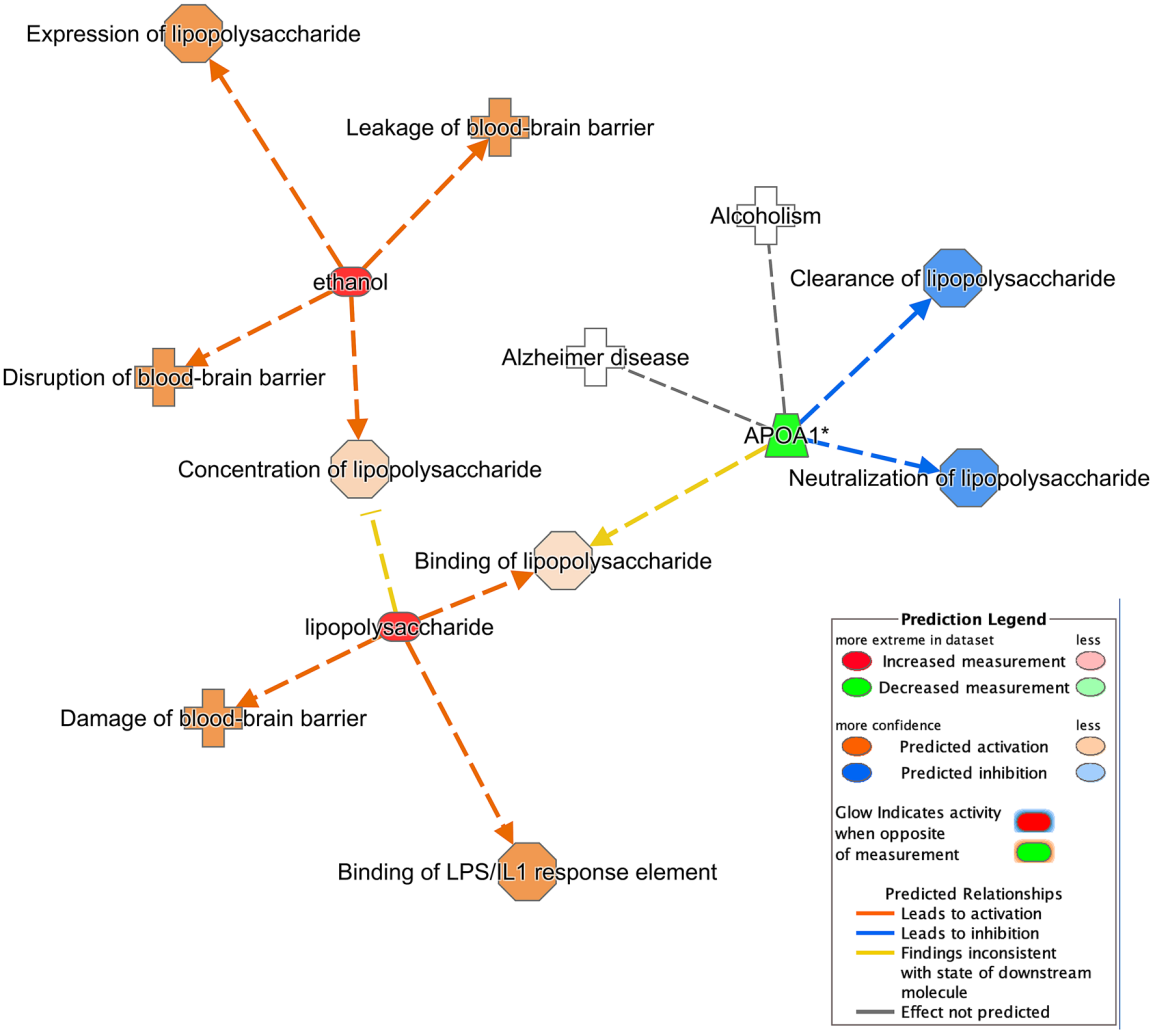
IPA analysis showing the relation of ethanol, LPS and the BBB. The orange line indicates activation; the blue line shows inhibition; and the yellow as inconsistent finding. The orange-filled shapes indicate predicted activation while the blue indicates predicted inhibition.

Figure 4 maps out the relationship between alcohol, AD, blood brain barrier (BBB) and lipopolysaccharide (LPS). Apolipoprotein A1 (APOA1) is a gene of interest that links alcoholism to Alzheimer’s Disease. The decreased level of APOA1 leads to the inhibition of clearance and neutralization of LPS, which is indicated by the blue molecules and the blue arrows. This figure also exhibits the effects of the alcohol increase and LPS in binge drinkers. In combination, the increase of both molecules leads to the disruption, damage and leakage of the BBB. The increase in ethanol also shows to activate the expression of LPS and the increase of LPS shows the activation of the LPS/IL1 response element and the LPS binding.

## Discussion

The oral cavity is not a homogenous environment as it contains multiple distinct environments for different microbial communities to colonize^33^. Currently, more than 700 prokaryotic taxa within the oral cavity have been detected, but most cannot be isolated and cultured through common methods^33^. To overcome this problem, one of the leading detection methods to study the oral microbiome is via 16 s ribosomal RNA (16 s rRNA) gene profiling, which is used in this study^33^. This study investigates the symbiosis state of a healthy oral microbiome, dysbiosis of binge drinkers, and how it relates to the pathogenesis of AD. Most studies focus on profiling the effects of alcohol on oral health in relation to the oral microbiome. This study, on the other hand, looks on two factors that are inter-related in the pathogenesis of AD, which are binge drinking and the oral microbiome. High levels of alcohol present in the oral cavity due to binge drinking may alter the oral environment and lead to oral microbiome dysbiosis. Alcohol can cause the reduction of salivary glands as well as the reduction of the oral mucosa based on a previously conducted study^20^. These changes lead to the overall change in the oral pH, which allows the microbiome to shift from a healthy microbiome to the pathogenic microbiome^34^. Despite being less studied compared to the gut microbiome, the oral microbiome has been linked to whole-body systemic diseases like rheumatoid arthritis and polycystic ovarian disease (PCOS)^10^. Early evidence from studies done at the University of Kentucky shows the baseline correlation between raised antibody levels in deficient cognitive ability and specific anaerobes of the oral microbiome, which are: *Fusobacterium nucleatum* (*F. nucleatum*) and *Prevotella intermedia* (*P. intermedia*)^35^. Both *F. nucleatum* and *P. intermedia* are dental pathogens that cause periodontist and produce LPS^36–38^.

Oral microbiome dysbiosis especially the decrease of *Firmicutes* by 4.51% in binge drinkers is significant, as this phylum has shown to be one of the dominant phyla in healthy oral microbiomes. The decrease of certain species like *Streptococcus* spp. and *Lactobacilli* has been detected in cancer patients^39^. A key insight indicating the significant findings of this study is that in 2017, a study by Vogt et al. has also found that *Firmicutes* phyla decreases are detected in AD patients in the gut microbiome^40^. They have also made the same observation where there is an increase in *Bacteroides* phyla, which makes up a diverse group of Gram-negative bacteria that produces LPS^40^. The similar observation among these findings indicates the correlation between oral microbiome dysbiosis in AD pathogenesis. The NIAAA defines moderate drinking as drinking up to one drink for women and two drinks for men per day^21^. The study by Fan et al. shows a decrease of *Firmicutes* phyla by 5.73% but an increase of the other phyla between 0.58% and 3.27%^28^. The most significant increase between moderate and binge drinkers is the *Fusobacteria* phyla*.* On the other hand, the most significant decrease observed in both moderate and binge drinkers is *Firmicutes* phyla. Similar findings in moderate and binge drinkers indicates that even in moderate amounts, alcohol plays a crucial role in altering the oral microbiome.

Bacterial endotoxin LPS can be found in almost all Gram-negative bacteria as it is a major component of its outer surface membrane component^41^. This study has found an increase of *Bacteroides* phylum in binge drinkers, which is a key overlap between binge drinkers and AD patients. A member of the *Bacteroides* phylum, *P. gingivalis,* is an important species as it is found to be significant in both of these two conditions along with being linked to the development of chronic periodontitis^37,42^. *P. gingivalis* links to AD and alcohol has been studied, but the correlation between the three has never been established. In terms of AD, *P. gingivalis* and other caries-producing bacteria are able to initiate innate immune signaling pathways via TLR-2 and TLR-4, which causes the release of cytokine like IFN-α and TNF-α. These cascading reactions will ultimately lead to the change of the BBB permeability that will cause vital cellular function to be destroyed, causing irreversible damage to the neurons^38,43^. *Neisseria meningitidis* is a known pathogen that causes inflammation of brain meninges which could lead to long term cognitive impairment^44^. The production of LPS in Gram-negative bacteria may also lead to the pathogenesis of AD^45,46^.

The activation of eIF2, regulation of eIF4, p70S6K signaling and mTOR due to binge drinking have all been linked to the pathogenesis of AD^30,47,48^. All three pathways are found to be significant via IPA in Fig. 2. In an in vivo study, LPS has caused the ER stress and has also caused the activation of the eIF2 pathway^49,50^. In eukaryotic cells, the highly conserved eIF2 signaling pathway plays a vital role in regulating cytokine expression during bacterial invasion^50^. In chronic alcoholic adult hippocampus, eIF2 signaling is involved in the clearance of ER stress. If this stressor cannot be removed, cellular apoptosis will occur^51^.

Currently, there is no data linking the oral microbiome to the eIF2 signaling pathway, but there is evidence of the two seen in the gut microbiome^52^. As seen in Fig. 3**,** six molecules detected in the eIF2 pathway are linked to both binge drinkers and AD. This finding suggests a strong correlation between the eIF2 pathway in binge drinkers and the pathogenesis of AD via the oral microbiome. Currently, there are no studies on linking the alteration of the oral microbiome due to binge drinking as a marker for AD. Based on data obtained from the different studies of the gut microbiome, however, a microarray analysis of mice shows the mice gut microbiome controls microglia maturation and function via eIF2^52^. Microglia plays an important role in the pathogenesis of AD either from genetic, age or environmental effects. As the β-amyloid accumulates, microglia becomes nonconstructive, which causes it to eat synapses and begin to secrete neurotoxic cytokines that cause injury to the neurons, leading to neurodegeneration^52^. Based on the results of the IPA analysis of the eIF2 pathway, there is an increased level of PKR in binge drinkers along with its detection in AD patients. PKR is a strong candidate for the next step in this study as ethanol consumption has shown to cause stress on the ER, which triggers the activation of the eIF2α, PERK and PRK^53^. Bacterial infection can trigger the eIF2 signaling pathway due to the presence of LPS in the cell, which can cause ER stress^50^. ER stress due to LPS has been documented in *Chlamydia trachomatis* infections, which in turn activates PKR^54^. Aside from LPS, the presence of bacterial RNA modulating PKR activation has been observed in cardiac tissue, but no study has been done to observe the effects on any neurological tissue^55^. This suggests that PKR is a good candidate for further investigation to characterize the pathogenesis of AD due to the activation of the eIF2 pathway in the presence of elevated LPS and ethanol.

Figure 4 shows that the increased level of ethanol increases the expression of LPS and the increased level of both ethanol and LPS leads to the prediction of the activation of multiple BBB diseases, including the disruption, leakage and damage towards the BBB. BBB, which consists of brain endothelial cells and perivascular mural cells, functions as a protective layer that maintains the hemostasis of the CNS by forming a tight control that regulates the entry of neurotoxic plasma-derived protein, metals, red blood cells, leucocytes and pathogens^56^. BBB damage among AD patients is widely seen in post-mortem studies, which include imaging scans that show microbleed and the accumulation of iron in patients^56^. The close proximity of the oral cavity to the BBB is a key interest as it provides a closer spatial and temporal study site as compared to the gut. During a bacterial infection, the production of proinflammatory cytokine may lead to a prolonged exposure of the BBB to the cytokine, which compromises the integrity of the BBB^27^. The dysbiosis of the oral microbiome in the presence of elevated levels of ethanol may further weaken the BBB, thus leading to the pathogenesis of AD. IPA also found a decrease in APOA1 concentration in both AD and alcoholism, which leads to the decrease of LPS clearance and neutralization. APOA1 can be found typically in the liver, intestines and the cerebral endothelial cells and is involved in the cellular cholesterol metabolism^57^. APOA1 is another good candidate that correlates binge drinker’s oral microbiome to AD as the lower level of APOAI has been documented in the gut microbiome analysis of patients with traumatic spinal cord injury^58^. IPA found inconsistencies with downstream molecules for the binding of LPS when APOA1 is decreased. This is expected as IPA is a knowledge base that compiles information and since the microbiome field is still within its infancy stage compared to other microbiology fields, not much data is available to create a high-level prediction of relationship between these two.

The relationship between alcohol, oral microbiome and AD (especially in the BBB) can be studied in further detail via single cell RNA sequencing (scRNA-seq). The single cell level response due to the alteration of the oral microbiome in the presence of alcohol can provide insight into the continuous cellular response and mechanism that may lead to the permeability of BBB and eventually AD^59^. This technique has been used to study the effects of LPS in neuroinflammatory responses and also 2 different AD studies^60–62^. By using scRNA-seq, the distinct types of neurons, glial cells and microglia can be precisely identified by this change^61^. This method can also be used to further profile the diversity of the oral cavity as seen in the study done on the effects of koumiss on the oral microbiome^63^. This study shows the potential of increasing the detection limit, thus leading to a more precise analysis in recognizing the change in diversity in the oral microbiome.

The increase in microbiome study shows how the forgotten endocrine organ plays a vital role in the host system. The diversity of the oral microbiome microenvironment along with its close proximity to the CNS makes it a good candidate for studying the pathogenesis of different neurological diseases. This study suggests there is a correlation between oral microbiome dysbiosis due to alcohol and AD. Since the composition of the oral microbiome can be altered due to binge drinking, it creates a link between ethanol and the oral microbiome to the pathogenesis of AD.

This study analyzes the effect of binge drinking on the oral microbiome and how it relates to the development of AD. This study also suggests that two genes, PKR and APOA1, are upregulated and downregulated in this study. More studies need to be carried out to further understand the role of PKR and APOA1 in the pathogenesis of AD due to oral microbiome dysbiosis from binge drinking.

## Materials and methods

### Oral microbiome

 Oral microbiome data has been obtained from Sequence Read Archive (SRA) with the accession number SRP133146 and SRP133149^28^. The data is cataloged under the umbrella BioProject PRJNA434300 and PRJNA434312, which focuses on the association of the oral microbiome with neck, head, and pancreatic cancer. The data source has been chosen with the focus on the association between oral microbiome and alcohol. The oral microbiome associated with AD has been found through the Disbiome Database, a database managed by Ghent University that curates the microbial composition and associates it with different diseases along with information publicly available through PubMed^29^.

### Data source

**Figure 5:**
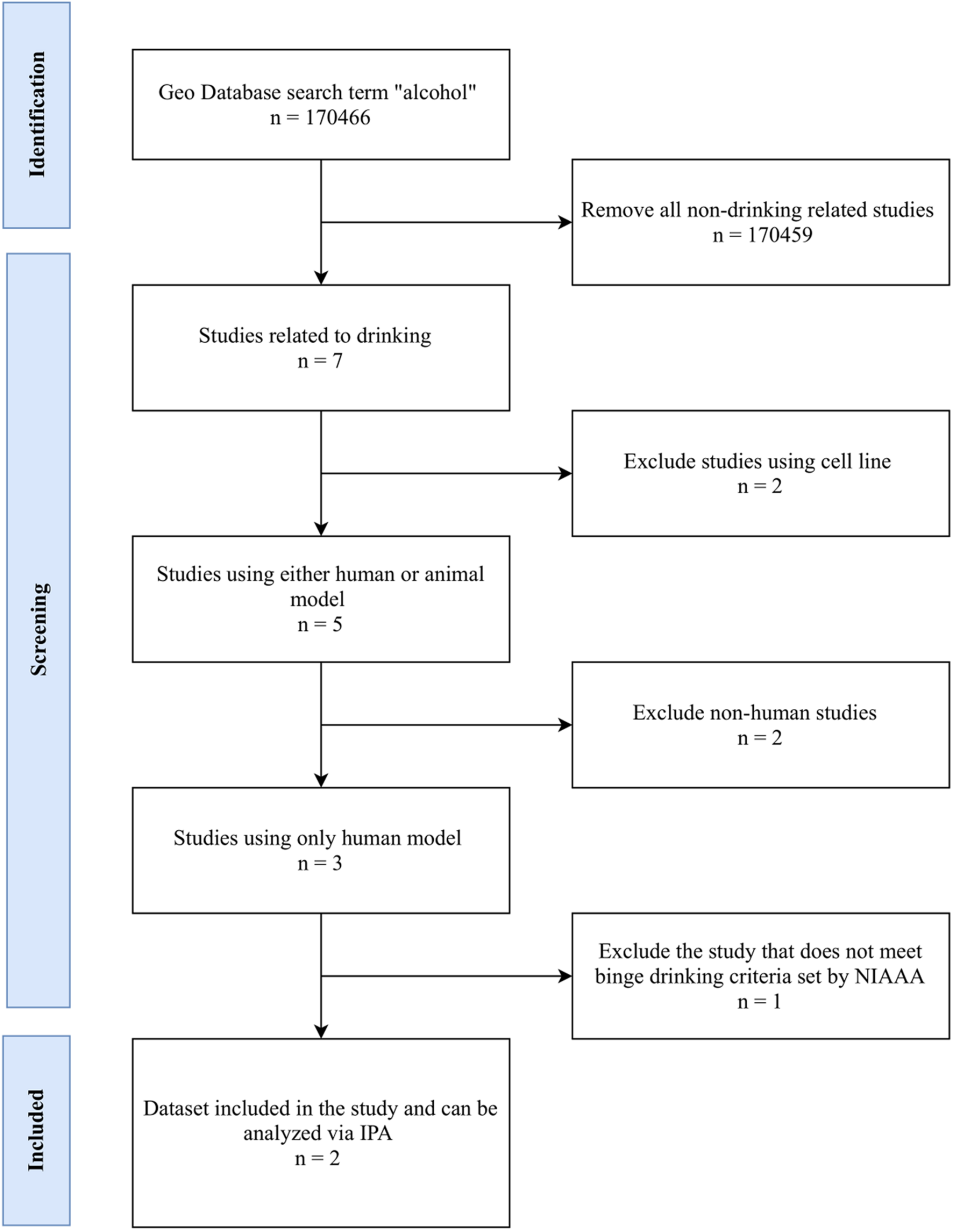
GEO data flow diagram. The experimental design is based on the recommendation provided by PRISMA. The method of exclusion is described in the material and method section.

The data has been obtained via Gene Expression Omnibus (GEO) databases using the key term “alcohol”. The search has been done based on the flow chart in Fig. 5 with the guidelines provided by Preferred Reporting Items for Systematic Reviews and Meta-Analyses (PRISMA)^64^. The PRISMA guideline can be found through the official website: https://www.prisma-statement.org/Protocols/. The guideline provides 27 itemized sections for the documentation and reporting of a systemic review and meta-analysis^64,65^. Among them, 17 items from the checklist have qualified to be used in this study.

Seven studies have been identified and screened for this study. Five of the studies have been excluded from this analysis based on the following criteria: exclude all cell culture samples, exclude all non-human samples, and exclude studies that do not meet binge drinking criteria. According to the NIAAA, binge drinking is consuming four or more drinks for women and five or more drinks for men on one occasion, or the blood alcohol concentration of 0.08%. Two studies fit these criteria. The accession number for these two studies are PRJNA190012; GSE44456^51^ and PRJNA 94069; GSE3846^66^. Table 1 shows the demographics of the samples used in this study. A total of 19 controls (6 females, 13 males) and 20 alcoholics (6 females, 14 males) from GSE44456; 6 controls and 6 alcoholics from GSE3846 are analyzed. The GSE3846 study does not provide demographic information in their study. Figure 5 shows the flow chart based on PRISMA guidelines and the data has been analyzed via IPA^67^.

**Table 1:** Demographics of the samples that have been used for this study. The table shows the demographics for PRJNA190012; GSE44456^51^.

	Control		Binge drinker	
Age	Female	Male	Female	Male
30–39	0	0	0	1
40–49	1	2	1	2
50–59	2	7	2	7
60–69	1	3	2	3
> 70	2	1	1	1
Total	6	13	6	14

### IPA analysis

QIAGEN Ingenuity Pathway Analysis (IPA) is used to observe the effects of binge drinking on the activation of different pathways. The biological effects of alcohol are analyzed via its Core Expression Analysis function and the results are based on signaling pathways and functional responses. The cutoff is set to 7 as this allows the maximum number of expressions. The canonical pathways with a *p* value below 0.05 are considered significant. The interactions among lipopolysaccharides (LPS), ethanol and binge drinking are analyzed via the Build a Pathway setting and Molecule Activity Predictor (MAP) overlay indicating the activation or inhibition of different conditions.

## Supplementary information

Supplementary Information.

## Abbreviations

NIH: National Institute of Health
HM: Human microbiome
HOMD: Human Oral Microbiome Database
CNS: Central nervous system
AD: Alzheimer’s disease
NIAAA: National Institute on Alcohol Abuse and Alcoholism
BAC: Blood alcohol concentration
AB: Amyloid beta
BBB: Blood brain barrier
eIF2: Eukaryotic initiation factor 2
mTOR: Mammalian target of rapamycin
eIF2α: Eukaryotic initiation factor 2 alpha
EIF2AK2 or PRK: RNA-activated protein kinase or eukaryotic translation initiation factor 2 alpha kinase 2
EIF2AK3 or PERK: Eukaryotic translation initiation factor 2 alpha kinase 3
ER: Endoplasmic reticulum
GDP: Guanosine diphosphate
GTP: Guanosine triphosphate
ATF4: Activating transcription factor 4
BACE1: β-Secretase enzyme
CREB: CAMP Response Element-binding Protein
LPS: Lipopolysaccharide
APOA1: Apolipoprotein A1
16s rRNA: 16S ribosomal RNA
PCOS: Polycystic ovarian disease
TLR2: Toll-like receptor 2
TLR4: Toll-like receptor 4
scRNA-seq: Single-cell RNA sequencing
SRA: Sequence Read Archive
GEO: Gene Expression Omnibus
PRISMA: Preferred Reporting Items for Systemic Reviews and Meta-analyses
